# Effects of Lavender Inhalation Aromatherapy on Depression in Individuals Undergoing Drug Treatment for Substance Dependence: A Randomized Clinical Trial

**DOI:** 10.1002/hsr2.72796

**Published:** 2026-08-05

**Authors:** Zahra Sotoudehnia Korrani, Amir Musarezaie, Mahtab Naraghirad, Meysam Rezazadeh

**Affiliations:** ^1^ Department of Occupational Health Engineering Sirjan School of Medical Science Sirjan Iran; ^2^ Department of Medical Surgical Nursing, School of Nursing and Midwifery Isfahan University of Medical Sciences Isfahan Iran; ^3^ Department of Geriatric Nursing, School of Nursing and Midwifery Shiraz University of Medical Sciences Shiraz Iran; ^4^ Nursing and Midwifery Care Research Center Isfahan University of Medical Sciences Isfahan Iran

**Keywords:** aromatherapy, depression, drug treatment, lavender, substance dependence, substance use disorder

## Abstract

**Background and Aims:**

Depression is common among individuals undergoing treatment for substance dependence and may adversely affect outcomes. This study determined the short‐term effect of lavender aromatherapy on depressive symptoms in individuals undergoing treatment for substance dependence.

**Methods:**

This single‐blind, parallel‐group randomized clinical trial was conducted in residential addiction treatment centers in Isfahan, Iran, in 2025. Using convenience sampling, 56 participants were allocated to intervention (*n *= 28) and control (*n *= 28) groups by computer‐generated block randomization with a 1:1 ratio. Allocation concealment was maintained with sequentially numbered, opaque, sealed envelopes. The intervention group received lavender inhalation aromatherapy twice daily for three consecutive days, plus routine treatment and care, whereas the control group received routine treatment and care only. Depression was measured using the DASS‐21 depression subscale before and immediately after the intervention. Data were analyzed using IBM SPSS Statistics version 26.

**Results:**

Baseline depression scores were not significantly different between groups (14.71 ± 5.50 vs. 13.11 ± 5.94; mean difference = 1.61, 95% CI: −1.46 to 4.67; Cohen's *d *= 0.28; *p *= 0.30). After the intervention, depression scores were lower in the intervention group than in the control group (5.11 ± 3.75 vs. 12.29 ± 6.17; mean difference = −7.18, 95% CI: −9.91 to −4.44; Cohen's *d *= −1.41; *p *< 0.001).

**Conclusion:**

Lavender inhalation aromatherapy was associated with a significant short‐term reduction in depressive symptoms among individuals undergoing drug treatment for substance dependence. Given the short intervention period, immediate assessment, absence of follow‐up, lack of placebo or attention control, and self‐report outcome measure, findings should be interpreted cautiously and not as definitive evidence of durable or specific clinical efficacy. Longer trials with follow‐up and appropriate comparators are needed.

## 1 Introduction

A large amount of money is spent annually on treating addicts, and thousands of people die from drug abuse [1]. Despite the dangers and complications of addiction, the number of victims of this deadly trap increases every day [2]. Considering the decreasing age of addiction, the human and material costs of combating the supply and distribution of drugs, the physical and psychological complications, the high time and cost, the low success rate, and the lack of motivation of addicts to recover, who seek drug treatment only to relieve their hangover, drug treatment has not yielded very good results for addicts [3]. Also, according to studies, 20%–90% of addicts who undergo drug treatment relapse [4]. A review of previous studies shows that the effectiveness of drug maintenance treatments without other interventions is not very successful due to the emphasis on drug use [2].

This is while patients with substance‐dependent disorders are also simultaneously affected by other disorders [5]. In addition to drug dependence disorder, these patients usually suffer from depression [6, 7]. Depressive disorders are considered to be comorbid addiction disorders [8]. On the other hand, a bidirectional relationship has been observed between these mental disorders and drug abuse, such that the use of these drugs causes depression and depression also encourages the person to use drugs [6]. Depression also affects the results of addiction treatment [9]. Therefore, drug dependence treatment should be carried out simultaneously with the treatment of depression and other disorders affecting general health to increase the success rate [6, 10].

Depression is evident at all stages of the life of patients with drug dependence disorders, including when they have used more than usual amounts or when they have symptoms due to withdrawal [11]. The occurrence of depression following drug withdrawal is one of the most obvious symptoms of addiction, so among the psychiatric disorders associated with addiction, depression is of particular importance because the loss of energy and hopelessness caused by depression can weaken the motivation of patients with drug‐dependent disorders to quit and seek treatment. It can also cause high‐risk behaviors such as suicide attempts and self‐harm [12]. Depressive symptoms are common in patients with drug‐dependent disorders. About one‐third to one‐half of those who abuse or are dependent on drugs have met the diagnostic criteria for major depressive disorder at some point in their lives [13]. On the other hand, some studies have shown that addicts undergoing methadone maintenance treatment have a high level of mental health problems compared to normal individuals. For example, Peles et al. showed in their study that most addicts undergoing methadone maintenance treatment experience psychological (mood) disorders such as depression [14]. Carpentier et al. showed in their study on 193 patients undergoing methadone maintenance treatment that 78% of them had psychiatric problems and needed further interventions [15]. Narcotics can lead to addiction in the short term by eliminating annoying or unpleasant states such as pain, depression and insomnia, while all these effects are temporary and in the long term, continued use of narcotics leads to increased depression, sleep disorders and resistance to pain, which ultimately leads to an increase in the dose consumed and complications such as increased depression in addicts in a vicious cycle [16].

To treat, control or modify depression in drug‐dependent groups, many strategies have been recommended by researchers, but in recent years, the use of complementary medicine has received special priority in this field. One of the complementary medicine methods that nurses directly intervene in when needed is aromatherapy [17]. Aromatherapy is used as a part of nursing care in many countries and is the second most commonly used complementary medicine treatment method among nurses in the clinic [18]. Aromatherapy has been proposed as a simple, accessible and non‐invasive technique that does not require any special equipment. Due to its simple and safe implementation, it has attracted the attention of many people [19]. Many medicinal plants, including lavender, are used in aromatherapy [20]. Lavender essential oil is one of the most common volatile oils used in aromatherapy [21]. Lavender is a member of the mint family and is used in traditional medicine for conditions such as anxiety, fatigue, arousal, headache, forgetfulness, depression, colds, and digestive and liver diseases [22].

Although the findings of some studies indicate the positive effects of lavender aromatherapy in reducing depression, for example, the study by Reven et al. showed significant effects of aromatherapy in reducing depression and anxiety in adults undergoing treatment for substance use disorder [23], the results of studies on the effects of aromatherapy on depression are contradictory. Among the studies that contradict the effect of aromatherapy on depression, the study by Tayebi et al. can be mentioned. The results of this study showed that lavender aromatherapy was not statistically significant on depression in hemodialysis patients [24]. In line with this result, in another study, the findings of Hata et al. indicated that aromatherapy was not statistically significant on depression in smokers during smoking cessation treatment and further studies are needed [25].

Considering factors such as the existence of contradictory studies on the effect of lavender aromatherapy on depression in different study populations, and considering that in the studies conducted, researchers did not find a study that investigated the effect of lavender aromatherapy on depression in the target population, namely addicts undergoing drug treatment, this doubles the importance of conducting the present study. Since disorders such as depression play an important role in relapse to substance use and most studies indicate a strong relationship between substance abuse and psychological disorders such as depression [26], measures beyond routine treatments seem necessary. Thus, since the co‐occurrence of depression can affect the treatment of individuals with addiction, study findings indicate the benefit of simultaneous treatment of substance abuse and mental disorders in addicted individuals [6]. Therefore, this study was conducted to determine the effect of lavender inhalation aromatherapy on depression in addicts undergoing drug treatment.

## 2 Methods

### 2.1 Study Design and Setting

The present study was a single‐blind, parallel‐group, randomized controlled clinical trial with two groups: an intervention group and a control group. The study was conducted in selected residential addiction treatment centers in Isfahan, Iran, in 2025. The study was reported in accordance with the CONSORT guideline for randomized controlled trials [27].

### 2.2 Eligibility Criteria

Inclusion criteria included age 18 years and older, ability to read and write, willingness and consent to participate in the study, no history of allergic rhinitis, eczema, or known respiratory problems such as asthma, no cold or any other condition that affects the individual's ability to smell, positive olfactory test (ability to identify the odor of the desired aromatherapy solution) as self‐reported, no simultaneous participation in another similar study, no history of lavender allergy (cutaneous, inhaled, or contact) as self‐reported, and no history of nasal drug use (sniffing). Exclusion criteria included missing more than one aromatherapy session, death of the participant during the study, and symptoms of lavender allergy (skin irritation, respiratory problems, eye irritation, gastrointestinal problems such as vomiting and diarrhea) during the study.

### 2.3 Sampling and Sample Size

**Figure 1:**
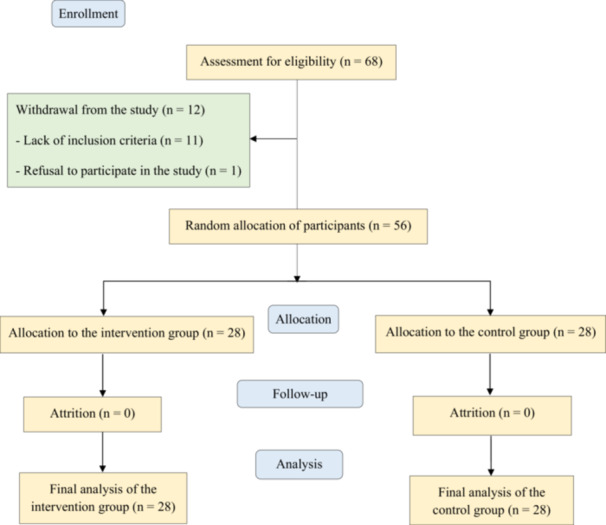
CONSORT flow diagram of participant enrollment, allocation, follow‐up, and analysis.

Eligible participants were recruited from the study population using convenience sampling and based on the eligibility criteria. After enrollment and baseline assessment, participants were randomly assigned to the intervention and control groups using a computer‐generated block randomization sequence with a 1:1 allocation ratio. To calculate the sample size in the present study, the following formula for comparing two independent means and the study of Reven et al. were used [23]: $n=\left[{\left(Z_{1-\alpha /2}+Z_{1-\beta }\right)}^{2}\times \left({S_{1}}^{2}+{S_{2}}^{2}\right)\right]/{\left({\bar{X}}_{1}-{\bar{X}}_{2}\right)}^{2}$. Based on this calculation, 25 participants were required for each group. Considering a 10% attrition rate, the sample size was increased to 28 participants per group, resulting in a total sample size of 56 participants (Figure 1).

### 2.4 Randomization and Blinding

An independent statistician generated the random allocation sequence using a computer‐based random number generator. Block randomization with a fixed block size of 4 and a 1:1 allocation ratio was used to ensure balanced group sizes throughout the recruitment process. Each block contained two assignments to the intervention group and two assignments to the control group.

To maintain allocation concealment, the allocation sequence was placed in sequentially numbered, opaque, sealed envelopes (SNOSE), which were prepared in advance by an independent person not involved in participant recruitment, intervention delivery, or outcome assessment. The principal investigator enrolled participants, obtained written informed consent, and conducted baseline assessments. After completion of baseline data collection, the next envelope in sequence was opened to reveal the participant's group assignment. Therefore, group allocation was concealed from the recruiter and the participant until enrollment and baseline assessment had been completed.

Blinding was considered at the levels of participants, researchers/intervention providers, and data analysts. Due to the recognizable odor of lavender essential oil and the absence of a sensory‐matched placebo comparator, blinding of participants after group allocation was not feasible. In addition, blinding of the researcher/intervention provider was not possible because the intervention required direct preparation and administration of lavender essential oil. However, data analysts were blinded to group allocation until completion of the primary analysis. Therefore, the study was conducted as a single‐blind randomized clinical trial, with blinding applied at the level of data analysis.

### 2.5 Data Collection Tools

The data collection tools in this study included a demographic questionnaire to collect personal information and disease characteristics (age, age at onset of drug use, employment status, education, marital status, kind of substances used, income status, number of times of quitting drugs, and duration of drug use) and a standard questionnaire (DASS‐21) to assess depression. The validity and reliability of this tool have been evaluated and confirmed many times. This tool has also been confirmed in Iran and has been used in various studies [28, 29, 30]. By comparing this questionnaire and psychiatric diagnostic interviews, its sensitivity and specificity for diagnosing depression were calculated to be 89%. Cronbach's alpha of this tool for depression was 0.77 in the Persian version. Its correlation coefficient with Beck's Depression Test was also mentioned as 0.7 [30]. Also, in the present study, to ensure the acceptable reliability of this questionnaire in the population of addicts undergoing drug treatment, using Cronbach's alpha method, Cronbach's alpha coefficient for the entire questionnaire was calculated as 0.85 and for the depression subscale as 0.90 in a pilot study, which indicates the desirable reliability of this questionnaire.

### 2.6 Intervention Procedure

The samples were entered into the study on the second night after admission. Before entering the study, patients were evaluated for lavender allergy by first asking the patient whether they had a history of any allergy or intolerance symptoms when consuming lavender in any form (inhalation, topical application to the skin, or oral administration). In the next step, a small amount of lavender essential oil was applied topically to the arm (middle third of the forearm) and monitored for local allergic reactions such as skin irritation, redness, inflammation, or itching, as well as systemic symptoms such as respiratory problems, eye irritation, and digestive problems within 24 h. The intervention group underwent lavender aromatherapy in addition to receiving drug treatment and routine care. In the intervention group (aromatherapy), the participants in the study underwent lavender aromatherapy for 3 days at 10 a.m. and 6 p.m. The researcher dripped two drops of 100% pure lavender essential oil (product of Barij Essential Oil Company, Kashan in Iran) with a dropper onto a 10 × 10 cm gauze and attached it to the participant's collar 20 cm from the nose with a clip. After that, the patient was asked to breathe normally for 20 min [31, 32]. Aromatherapy was performed for patients in the intervention group for 20 min; their depression levels were measured immediately before and after the end of the intervention. The intervention was conducted in the residential treatment center under similar routine ward conditions for all participants. Aromatherapy sessions were performed at fixed times of the day, and participants remained in the treatment setting during the intervention period. The intervention and control groups were hospitalized in separate wards to reduce contamination between groups and co‐exposure to lavender odor. Participants' attendance at the scheduled aromatherapy sessions was used to verify adherence to the intervention protocol, and missing more than one aromatherapy session was considered an exclusion criterion. No chemical composition analysis of the lavender essential oil was performed in the present study. The control group, hospitalized in another ward, received routine treatments and care, and their depression levels were assessed using questionnaires at the same time intervals as the intervention group.

### 2.7 Ethical Considerations

This study was approved by the Ethics Committee of Isfahan University of Medical Sciences (ethics approval code: IR.MUI.NUREMA.REC.1403.185) and was registered in the Iranian Clinical Trials Registry (registration code: IRCT20141127020108N9). After obtaining the necessary permissions, the researchers entered the sampling setting. Eligible participants were informed about the purpose and procedures of the study, voluntary participation, the right to withdraw at any time, confidentiality of information, and that participation was free of charge. Written informed consent was obtained from all participants before enrollment. The demographic information form was then completed through interviews with the participants.

### 2.8 Data Analysis

Statistical analyses were performed using IBM SPSS Statistics version 26 (IBM Corp., Armonk, NY, USA). Statistical analyses were conducted and reported in accordance with standard recommendations for clinical research statistics and the SAMPL guidelines [33]. Continuous variables were summarized as mean ± standard deviation (SD), and categorical variables were reported as number and percentage. The normality of continuous variables was assessed using the Kolmogorov–Smirnov test.

The primary pre‐specified analysis was the comparison of depression scores between the intervention and control groups and within each group before and after the intervention. Baseline demographic and substance‐use‐related variables were also compared between groups to assess group comparability. No exploratory subgroup analyses were conducted. An independent‐samples *t*‐test was used to compare normally distributed continuous variables between the two groups. A paired‐samples *t*‐test was used to compare within‐group changes in depression scores before and after the intervention. *χ* ^2^ test or Fisher's exact test, as appropriate, was used to compare categorical variables between groups. To further account for baseline depression scores, a baseline‐adjusted sensitivity analysis was conducted using analysis of covariance (ANCOVA), with post‐intervention depression score as the dependent variable, study group as the fixed factor, and baseline depression score as the covariate. The homogeneity of regression slopes assumption was assessed by testing the interaction between study group and baseline depression score. All randomized participants completed the study and were included in the final analysis according to their originally assigned groups; therefore, no imputation for missing outcome data was required.

Effect sizes and 95% confidence intervals (CI) were calculated and reported where appropriate to indicate the magnitude and precision of the observed differences. The a priori level of statistical significance was set at 0.05. All statistical tests were two‐sided. *p* values were reported according to standard reporting conventions.

## 3 Results

The results of the independent‐samples *t*‐test showed no statistically significant difference in age between the intervention group (43.39 ± 14.12 years) and the control group (43.75 ± 13.14 years) (*p *= 0.92). Other information regarding the number of previous quit attempts and the duration of drug use is presented in Table 1. In addition, the results of the *χ* ^2^/Fisher's exact test showed no statistically significant differences between the intervention and control groups in demographic and substance‐use‐related variables, including age at onset of drug use, employment status, education level, marital status, type of substance used, and income status (Table 2; *p *> 0.05).

**Table 1:** Comparison of quantitative demographic and abuse variables between the intervention and control groups in patients undergoing drug treatment.

Variables	Groups	Mean ± SD^a^	*p* value^b^
Age	Intervention	43.39 ± 14.12	0.92
	Control	43.75 ± 13.14	
Number of times of quitting drugs	Intervention	9.93 ± 7.98	0.74
	Control	10.68 ± 8.98	
Duration of drug use	Intervention	19.57 ± 14.00	0.76
	Control	18.25 ± 15.14	

a Standard deviation.

b Independent *t*‐test.

**Table 2:** Comparison of qualitative demographic and abuse variables between the intervention and control groups in patients undergoing drug treatment.

Variables	Groups	Control number (percentage)	Intervention number (percentage)	*p* value
Age of onset of drug use	Childhood and adolescence	10 (35.70)	14 (50.00)	0.55^a^
	Youth	11 (39.30)	9 (32.10)	
	Middle age and old age	7 (25.00)	5 (17.90)	
Employment status	Unemployed	10 (35.70)	12 (42.90)	0.94^b^
	Employee	5 (17.90)	3 (10.70)	
	Freelancer	10 (35.70)	10 (35.70)	
	Retired	3 (10.70)	3 (10.70)	
Education level	Elementary	4 (14.30)	6 (21.40)	0.73^a^
	Middle school	6 (21.40)	7 (25.00)	
	Diploma	11 (39.30)	11 (39.30)	
	Academic	7 (25.00)	4 (14.30)	
Marital status	Married	8 (28.60)	7 (25.00)	0.76^a^
	Single	20 (71.40)	21 (71.00)	
kind of substances used	Natural	11 (39.30)	11 (39.30)	> 0.99^a^
	Synthetic	17 (60.70)	17 (60.70)	
Income status	Dissatisfaction	12 (49.20)	15 (53.60)	0.42^a^
	Satisfactory	16 (57.10)	13 (46.40)	

a *χ* ^2^ test.

b Fisher's exact test.

In addition to *p* values, effect sizes and 95% confidence intervals were reported to indicate the magnitude and precision of the observed differences. In the intergroup comparison, before the intervention, the mean depression score did not differ significantly between the intervention and control groups (14.71 ± 5.50 vs. 13.11 ± 5.94; mean difference = 1.61, 95% CI: −1.46 to 4.67; Cohen's *d *= 0.28; *p *= 0.30). After the intervention, the mean depression score was lower in the intervention group than in the control group (5.11 ± 3.75 vs. 12.29 ± 6.17; mean difference = −7.18, 95% CI: −9.91 to −4.44; Cohen's *d *= −1.41; *p *< 0.001).

In the intragroup comparison, the depression score decreased in the intervention group from 14.71 ± 5.50 before the intervention to 5.11 ± 3.75 after the intervention (mean change = 9.61, 95% CI: 6.81–12.40; Cohen's dz = 1.33; *p *< 0.001). In contrast, no statistically significant change was observed in the control group (13.11 ± 5.94 before the intervention vs. 12.29 ± 6.17 after the intervention; mean change = 0.82, 95% CI: −2.84 to 4.49; Cohen's dz = 0.09; *p *= 0.65). The between‐group comparison of mean change scores also showed a greater reduction in depression scores in the intervention group than in the control group (mean difference in change = 8.79, 95% CI: 4.28–13.29; Cohen's *d *= 1.05; *p *< 0.001) (Table 3).

**Table 3:** Intra‐ and inter‐group comparison of mean depression scores between the intervention and control groups.

Variable	Group	Before intervention mean ± SD	After intervention mean ± SD	Within‐group mean change	95% CI for change	Effect size	*p* value^a^
Depression	Intervention (*n *= 28)	14.71 ± 5.50	5.11 ± 3.75	9.61 ± 7.21	6.81 to 12.40	Cohen's dz = 1.33	*p *< 0.001
Depression	Control (*n *= 28)	13.11 ± 5.94	12.29 ± 6.17	0.82 ± 9.45	−2.84 to 4.49	Cohen's dz = 0.09	*p *= 0.65
Between‐group comparison before intervention	—	Mean difference = 1.61	—	—	−1.46 to 4.67	Cohen's *d* = 0.28	*p *= 0.30
Between‐group comparison after intervention	—	—	Mean difference = −7.18	—	−9.91 to −4.44	Cohen's *d* = −1.41	*p *< 0.001
Between‐group comparison of change scores	—	—	—	Mean difference = 8.79	4.28 to 13.29	Cohen's *d* = 1.05	*p *< 0.001

Abbreviations: CI, confidence interval; SD, standard deviation.

a Paired *t*‐test was used for within‐group comparisons. Independent *t*‐test was used for between‐group comparisons.

In the baseline‐adjusted ANCOVA, the homogeneity of regression slopes assumption was met, as the interaction between study group and baseline depression score was not statistically significant (F(1, 52) = 0.16; *p *= 0.69). After adjustment for baseline depression score, the between‐group difference in post‐intervention depression scores remained statistically significant (F(1, 53) = 25.58; *p *< 0.001; partial eta squared = 0.33). The adjusted mean depression score was 5.25 in the intervention group and 12.14 in the control group. The adjusted mean difference between the intervention and control groups was −6.89 (95% CI: −9.62 to −4.16), indicating lower post‐intervention depression scores in the intervention group. This finding was consistent with the unadjusted between‐group comparison and the comparison of mean change scores.

## 4 Discussion

The results of this study showed that lavender inhalation aromatherapy was associated with a significant short‐term reduction in depressive symptoms among individuals undergoing treatment for substance dependence, such that the intervention group had lower depression scores than the control group immediately after the intervention. Consistent with the present findings, Reven et al. reported beneficial effects of aromatherapy on depression in adults undergoing treatment for substance use disorders [23]. In addition, Cho and Kim, in a meta‐analysis of 32 randomized controlled trials, reported evidence suggesting that aromatherapy may help reduce depressive symptoms [34].

Although very few studies have been conducted on the effect of aromatherapy on depression in individuals with substance dependence undergoing drug withdrawal or drug treatment, studies in other target populations consistent with the results of the present study can be cited. For example, the results of Ebrahimi et al. showed that inhaled aromatherapy with both lavender and chamomile oils helped reduce depression levels in community‐dwelling elderly people [35]. Also, studies reporting reduced depression in breast cancer patients [36], improved depressive symptoms in prison patients [37], reduced depression in patients with mood disorders [38], reduced depression in patients with acute coronary syndrome [39], and prevention of postpartum depressive symptoms [40], are among the studies that are consistent with the potential beneficial effects of aromatherapy on depressive symptoms.

Regarding contradictory findings in the target population, namely the effect of aromatherapy on depression in individuals with substance dependence undergoing drug withdrawal, drug treatment, or other related conditions, the research team did not find similar studies. However, some studies in other target populations can be cited in which aromatherapy failed to produce a significant reduction in depressive symptoms. For example, Hata et al. examined the effects of aromatherapy on smokers with depressive symptoms during smoking cessation treatment and reported that aromatherapy had no significant effect in this group [25]. Given the differences in study populations, intervention protocols, outcome measures, and clinical conditions, direct comparison with the present study is limited.

This study is among the first population‐specific exploratory trials to examine the effect of lavender inhalation aromatherapy on depressive symptoms in individuals undergoing treatment for substance dependence. Therefore, the findings may provide preliminary evidence for the potential use of lavender aromatherapy as a low‐cost, accessible, and easy‐to‐administer adjunctive intervention alongside routine care in this population. However, given the short duration of the intervention, the absence of follow‐up assessment, and the lack of a placebo or attention‐control group, the findings should be interpreted cautiously and should not be considered definitive evidence of durable or specific clinical efficacy.

A further important limitation of the present study was the short intervention period and the absence of follow‐up assessment. The intervention was conducted over three consecutive days, and depression scores were assessed immediately after completion of the intervention. Therefore, the findings should be interpreted as indicating a short‐term reduction in depression scores rather than evidence of a durable antidepressant effect. Because no follow‐up assessment was performed, it was not possible to determine whether the observed improvement persisted over time or whether it reflected a transient improvement in mood immediately after aromatherapy. The absence of follow‐up was mainly related to the short duration of stay in residential addiction treatment centers and the practical challenges of post‐discharge follow‐up in this population, including the possibility of treatment discontinuation, relapse, and loss to follow‐up, which could introduce substantial attrition bias. In addition, although a statistically significant reduction in depression scores was observed, the clinical significance of this change should be interpreted with caution, particularly in the absence of longer‐term follow‐up and clinically defined outcome thresholds. Future studies with longer intervention periods and follow‐up assessments are recommended to evaluate the durability and clinical relevance of lavender inhalation aromatherapy in this population.

Another limitation of this research is the limited number of supporting studies in the target population, which makes it difficult to make more accurate comparisons and provide a more comprehensive interpretation of the findings. In addition, the use of convenience sampling limits the external validity and generalizability of the findings. The sample size was relatively small, which may have limited the statistical power of the study and increased the risk of overestimating the observed effect size. Furthermore, all participants were male, which limits the generalizability of the findings to female individuals undergoing drug treatment for substance dependence. Therefore, the findings should be generalized to broader populations with caution. Future studies with larger and more diverse samples, including female participants, longer intervention periods, a greater number of sessions, and follow‐up assessments are recommended to provide stronger evidence regarding the effect of lavender inhalation aromatherapy on depressive symptoms in individuals undergoing drug treatment for substance dependence. Overall, the results should be viewed as an observed short‐term association between lavender inhalation aromatherapy and reduced depressive symptoms, rather than conclusive evidence of sustained therapeutic efficacy.

Another methodological limitation of the present study was the absence of participant and intervention‐provider blinding and the lack of a placebo or attention‐control group. Because lavender essential oil has a distinctive odor, blinding of participants and the researcher administering the intervention was not feasible. Moreover, selecting an appropriate placebo for aromatherapy studies is methodologically challenging, because an odorless comparator would not adequately mimic the sensory characteristics of lavender inhalation, while a scented comparator cannot be assumed to be completely inert. In addition, because the outcome was assessed using a self‐report instrument, expectancy and performance biases cannot be excluded. Therefore, the observed reduction in depression scores cannot be attributed solely to the specific effect of lavender essential oil and may partly reflect non‐specific contextual, sensory, or expectancy‐related effects. Future randomized trials should include placebo or attention‐control conditions, where feasible, to better isolate the specific effects of lavender inhalation aromatherapy.

In addition, some aspects related to technical reproducibility were not fully controlled or reported. The chemical composition of the lavender essential oil was not analyzed, and environmental factors such as room ventilation and possible co‐exposure to other odors were not quantitatively assessed. Although the intervention and control groups were hospitalized in separate wards and the sessions were conducted under routine ward conditions, uncontrolled environmental odor exposure cannot be completely excluded. Furthermore, concurrent antidepressant or psychiatric treatments and methadone dose stability were not specifically controlled or analyzed. These factors may have influenced depressive symptoms and should be more carefully monitored and reported in future studies.

## Author Contributions

Meysam Rezazadeh designed and conducted the study, collected the data, and drafted the initial manuscript. Amir Musarezaie contributed to the conceptualization of the study, methodology development, and initial drafting. Mahtab Naraghirad and Zahra Sotoudehnia Korrani conducted the statistical analysis and contributed to methodological planning and data interpretation. All authors reviewed and approved the final version of the manuscript. Meysam Rezazadeh had full access to all of the data in this study and takes complete responsibility for the integrity of the data and the accuracy of the data analysis.

## Funding

The authors have nothing to report.

## Ethics Statement

This study was scientifically approved and registered as a research project by the Vice Chancellor for Research of Isfahan University of Medical Sciences with scientific code 1403254. Before the intervention began, the study was approved by the Research Ethics Committee of Isfahan University of Medical Sciences with ethics code IR.MUI.NUREMA.REC.1403.185 and was registered in the Iranian Clinical Trials Registry with registration code IRCT20141127020108N9.

## Consent

The authors have nothing to report.

## Conflicts of Interest

The authors declare no conflicts of interest.

## Transparency Statement

The corresponding author, Meysam Rezazadeh, affirms that this manuscript is an honest, accurate, and transparent account of the study being reported; that no important aspects of the study have been omitted; and that any discrepancies from the study as planned (and, if relevant, registered) have been explained.

## Data Availability

The data that support the findings of this study are available from the corresponding author upon reasonable request.
